# Soluble expression of recombinant coagulation factor IX protein using *Escherichia coli*

**DOI:** 10.1016/j.bbrep.2024.101714

**Published:** 2024-04-18

**Authors:** Byoung-Hee Park, Hanool Yun, Hee-Jin Jeong

**Affiliations:** a Industry-Academia Cooperation Foundation, Hongik University, 2639 Sejong-ro, Sejong-si, 30016, South Korea; b Department of Biological and Chemical Engineering, Hongik University, 2639 Sejong-ro, Sejong-si, 30016, South Korea

**Keywords:** Hemophilia, Factor IX, Recombinant protein, Protein expression, *Escherichia coli*

## Abstract

Hemophilia B is a congenital bleeding disorder caused by factor IX (FIX) deficiency. Generation of recombinant FIX (rFIX) is required for detecting a Hemophilia B indicator, anti-FIX antibody. In this study, we described a method for producing recombinant FIX (rFIX) using *Escherichia coli*. We constructed a FIX-expressing plasmid without a fusion tag protein-encoding gene and produced rFIX as a soluble form within five days. Dose-dependent curve was obtained from ELISA using anti-FIX antibody, indicating that the rFIX can be used as an antigen to detect anti-FIX antibody with high affinity and sensitivity.

## Highlights

• Generation of a recombinant factor IX (rFIX) to compensate for detecting anti-FIX antibody in patients with hemophilia B.
• Expression of a soluble form of full-length rFIX with no fusion sequence.
• Confirmation of the binding efficiency of rFIX to anti-FIX antibody.

## 1 Introduction

Factor IX (FIX) is a plasma glycoprotein that plays an important role in blood coagulation and hemostasis. The FIXa-factor VIIIa complex forms the tenase complex, which converts factor X to factor Xa that is essential for the formation of a stable blood clot and the maintenance of hemostasis. FIX deficiency, or Hemophilia B, causes difficulties in blood clot formation, resulting in prolonged bleeding after injury or surgery. Measurement of the FIX amount in patients is essential and antibody against FIX has been used for detecting FIX. However, high-cost commercial antibody has been used and generation of a recombinant antibody is required. FIX protein is used as an antigen to generate a novel recombinant anti-FIX antibody. Recombinant FIX (rFIX) has been developed as a pharmacological strategy to compensate for FIX deficiency in patients with Hemophilia B to prevent or control bleeding incidents. Commercially available rFIXs from various sources and literature describing rFIX production strategies have provided useful information for improving the pharmaceutical efficacy and application of rFIX. Mammalian cells, such as human embryonic kidney (HEK) and Chinese hamster ovary (CHO) cells, have been used as host cells to produce rFIX as a drug for patients with FIX deficiency because of their ability to perform post-translational modifications (PTMs) that bacterial cells, such as *Escherichia coli*, are unable to perform. However, because the cost efficiency of *E. coli*-based protein production and its cell growth rate are higher than those of mammalian cells, rFIX production in *E. coli* has the potential to reduce production cost. Although PTMs are necessary when rFIX is used as a drug, rFIX without PTMs can be used as an antigen to generate anti-FIX antibodies. *E. coli*-based rFIX production was first reported by Lin et al., in 1987 [1]. However, they only produced FIX fragments, which consisted of 13 different regions of FIX. Moreover, they fused gene *10* of bacteriophage T7 to FIX fragments to upregulate protein expression and downregulate protein degradation. However, the gene *10*-FIX fusion proteins were expressed in insoluble forms. Eventually, they conducted tedious refolding for protein solubilization before using the proteins as antigens to generate antibodies. Finally, polyclonal antibodies were obtained from the rabbit serum. However, as the antibodies were generated using gene *10*-FIX fusion proteins as antigens, the polyclonal antibody detected not only FIX, but also the gene *10* protein, resulting in low specificity. In 1996, Lee et al. expressed glutathione S-transferase (GST)-conjugated FIX in *E. coli* [2]. They produced a fusion protein using GST as a carrier protein to purify FIX using a glutathione resin, resulting in a high purity of the purified fusion protein, as expected. However, because FIX was generated in a GST-fused form, its further application was limited without the separation of GST from FIX. Although the authors mentioned the isolation of GST from the fusion protein, the results regarding the isolation were not representative. Commercially available research-grade rFIX proteins were produced using *E. coli* from Sino Biological Inc. And Cusabio Inc.; however, these proteins were also generated as GST-conjugated fusion proteins.

In this study, we aimed to produce a soluble form of full-length rFIX with no fusion sequence in *E. coli* without any carrier protein. We describe the details for researchers who have a substantial interest in constructing rFIX and further use it in various applications, including as an antigen source for the generation of a recombinant anti-FIX antibody as an FIX inhibitor and for detecting FIX, and to develop a screen for anti-FIX alloantibody formation in patients with hemophilia B [3]**.**

## 2 Materials and methods

### 2.1 Materials

Oligonucleotides were obtained from Bionics (Seoul, Korea). KOD-plus-neo polymerase was obtained from Toyobo (Osaka, Japan). In-fusion enzyme kit was obtained from Takara (Siga, Japan). *E. coli* SHuffle T7 Express lysY was obtained from New England Biolabs (Seoul, Korea). TB medium was obtained from Sigma Korea (Seoul, Korea). Ni-NTA affinity beads was obtained from GE healthcare (NJ, USA). 96-well microplate (Medi-binding) was obtained from SPL (Gyeonggi-do, Korea). HRP-conjugated anti-His-tag antibody (105,327-MM02T-H) and rabbit anti-FIX antibody (11,503-R034) were obtained from Sino Biological (Beijing, China). HRP-conjugated anti-rabbit IgG antibody (LF-SA8002) was obtained from Abfrontier (Seoul, Korea). Other chemicals and reagents, unless otherwise indicated, were from Sigma Korea (Seoul, Korea).

### 2.2 Gene cloning

The human FIX-coding gene (NM_000133.4) was synthesized (LncBio, Seoul, Korea). The DNA was amplified via PCR using primers, p1 (5′-atgaatcacaaagtgcagcgcgtgaacatgatc-3′) and p2 (5′-ccctaatgatgatgatgatgatgagtgagctttgttttttcc-3′, underline indicates a His-tag sequence), and Takara Ex Premier (Takara, Siga, Japan) under the following conditions: 98 °C for 2 m; 35 cycles of 98 °C for 20 s, 60 °C for 20 s, and 68 °C for 1 m. The pCold-I vector was linearized via PCR using primers, p3 (5′-catcatcatcattagggatccgaattcaagc-3′, underline indicates a partial His-tag sequence) and p4 (5′-cactttgtgattcatggtg-3′), and KOD-plus-neo polymerase under the following conditions: 94 °C for 2 m; 35 cycles of 98 °C for 10 s, 56 °C for 30 s, and 68 °C for 2 m 35 s. The PCR products were ligated using an In-fusion enzyme kit. After transformation, a candidate vector was selected by enzyme digestion using *Xba*I, and the sequence was confirmed via Sanger sequencing, resulting in pCold-I::FIX-H, which is a FIX-expressing gene with a His-tag-coding sequence at the 5-terminus of the FIX-coding sequence. To construct a pCold-I::H-FIX, which is a FIX-expressing gene with a His-tag-coding sequence at the 3-terminus of the FIX-coding sequence, the human FIX-coding gene was amplified via PCR using primers, p5 (5′-gaaggtaggcatatgcagcgcgtgaacatg-3′) and p6 (5′-gaattcggatccctaagtgagctttgttttttcc-3′, underline indicates a stop codon), and Takara Ex Premier under the following conditions: 98 °C for 2 m; 35 cycles of 98 °C for 20 s, 60 °C for 20 s, and 68 °C for 1 m. The PCR product was inserted into the pCold-I vector, which was linearized using primers, p7 (5′-tagggatccgaattcaagc-3′, underline indicates a stop codon) and p8 (5′-catatgcctaccttcgat-3′), and KOD-plus-neo polymerase under the following conditions: 94 °C for 2 m; 35 cycles of 98 °C for 10 s, 56 °C for 30 s, and 68 °C for 2 m 35 s, using the In-fusion enzyme.

### 2.3 Recombinant protein production

SHuffle T7 LysY cells were transformed using pCold-I::FIX-H or pCold-I::H-FIX, and at 37 °C for overnight in LBA medium (LB medium containing 100 μg/mL ampicillin) and 1.5 % agar. A single colony was grown at 37 °C in 4 mL of LBA medium until the OD_600_ reached 0.9 and subsequently inoculated at 37 °C 100 mL of 100 μg/mL ampicillin-containing TB medium. When OD_600_ reached 0.9–1.0, 0.05 mM isopropylthio-β-galactopyranoside (IPTG) was added, and further incubated for 72 h at 16 °C. Culture supernatants were clarified by centrifugation (3500 rpm, 20 min, 4 °C) and purified as follows: the supernatant was bound to 100 μL of Ni-NTA affinity beads for 1 h at room temperature (RT) and washed three times using 5 mL of washing buffer (50 mM phosphate, 0.3 M NaCl, 20 mM Imidazole, pH 7.4). Next, 3 mL of elution buffer (50 mM phosphate, 0.3 M NaCl, 0.5 M imidazole, pH 7.4) was added and reacted for 1 h at RT. 20 % glycerol was added to the eluent, and the protein was stored at −20 °C. The purity and concentration of the purified protein was confirmed by sodium dodecyl sulphate–polyacrylamide gel electrophoresis (SDS–PAGE) under reducing condition. The purified protein was reduced by mixing with 100 mM dithiothreitol-supplemented loading buffer (250 mM Tris–HCl, 10 % SDS, 50 % glycerol, and 0.05 % bromophenol blue, pH 6.8), followed by boiling at 95 °C for 5 min. The concentration of purified FIXs was quantified by SDS-PAGE using bovine serum albumin (BSA) standards. We measured the thickness of the target band for FIX with a size of 53 KDa using the ImageLab software (Bio-Rad) and converted the thickness to concentration by fitting the BSA titration curve, which was constructed using the thickness of the BSA band, the concentration of which was known.

### 2.4 Enzyme-linked immunosorbent assay

We conducted ELISA assays by immobilizing FIX-H or H-FIX in the wells of a 96-well plate, followed by incubation with a commercially available polyclonal anti-FIX IgG as the primary antibody (produced by immunizing rabbits to rFIX (NP_000124.1)), and an HRP-conjugated anti-rabbit IgG polyclonal antibody as the secondary antibody. Serially diluted FIX-H or H-FIX in PBS was immobilized to the 96-well microplate (Medi-binding) overnight at 4 °C. The plate was blocked with PBS0.05T3B (PBS buffer containing 0.05 % Tween-20 and 3 % BSA) for 2 h at RT. After washing with PBS0.05 T three times, 5000-fold diluted rabbit anti-FIX polyclonal IgG was added and incubated for 1 h at RT. Next, the plate was washed thrice using PBS0.05 T and incubated with 20,000-fold diluted HRP-conjugated anti-rabbit IgG for 1 h at RT. The plate was washed three times with PBS0.05 T and developed with TMB solution (TCI, Tokyo, Japan). After incubation for 10 min, the reaction was stopped by adding 1 N sulfuric acid and the absorbance at 450 nm was measured. Dose−response curve was constructed using the GraphPad Prism software (GraphPad Software, San Diego, CA). The EC_50_ value was calculated from the curve ﬁtting to a 4-parameter logistic equation of the software: Y = Bottom + (Top − Bottom)/(1 + 10 ((LogEC_50_-X) × Hill Slope)). The limit of detection (LOD) value was calculated based on the following equation: LOD = mean blank + 1.645 × SD_blank_ + 1.645 × SD_low concentration sample_ [4]. SD = standard deviation.

## 3 Results

### 3.1 Generation of recombinant FIX proteins

**Fig. 1:**
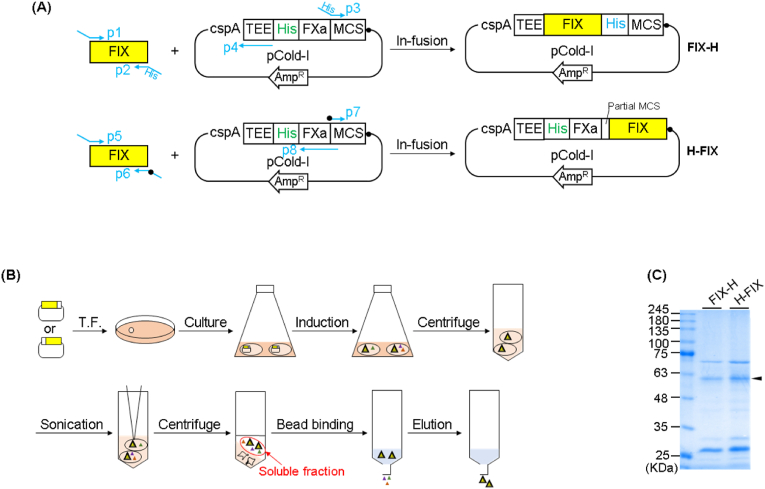
(A) Plasmid DNA maps for the expression of C-terminal His-tag-conjugated FIX (FIX-H) and N-terminal His-tag-conjugated FIX-coding gene (H-FIX). P, TEE, His (H), FXa, MCS, dot, and AmpR indicates primer, translation enhancing element sequence, His-tag, Factor Xa sequence, multiple cloning site, stop codon, and ampicillin resistance gene, respectively; (B) Schematic representation of the entire protein production system; T.F. indicates transformation; (C) SDS-PAGE analysis of FIX-H or H-FIX. An arrow indicates the location of target protein.

We first constructed an FIX-H-expressing plasmid (pCold-I::FIX-H) by amplifying the human FIX-coding gene (NM_000133.4) with a His-tag-coding sequence at the 5-terminus of the FIX-coding sequence to allow Ni-NTA-based protein purification. To construct an H-FIX-expressing plasmid (pCold-I::H-FIX), we inserted the coding sequence for the His-tag at the 3-terminus of the FIX-coding sequence (Fig. 1A). We next expressed each type of tagged protein by transforming *E. coli* cells using the C- or N-terminal His-tag-conjugated FIX-coding gene, and the culture supernatants were purified using Ni-NTA affinity beads (Fig. 1B). Our SDS-PAGE results confirmed that the target protein was the expected size (53 kDa) and was successfully expressed in a soluble form (Fig. 1C). When we expressed FIX-H and H-FIX on a 100 mL culture scale, 20 μg and 42 μg of purified protein were obtained, respectively.

**Fig. 2:**
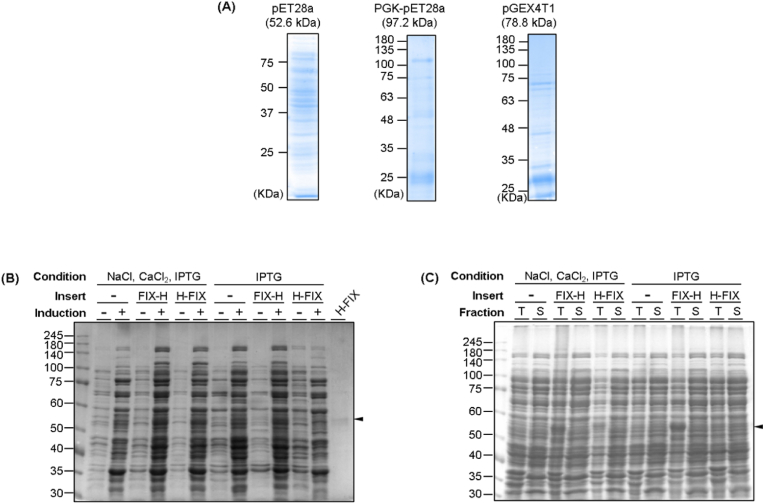
(A) SDS-PAGE analysis of the pET28a-, PGK-conjugated pET28a-, or GST-conjugated pGEX4T1-coded FIX proteins; (B) SDS-PAGE analysis of rFIX proteins and empty pCold-I-coded protein (a pCold-I vector without inserting an FIX-expressing gene) before or after induction; (C) SDS-PAGE analysis of total (T) or soluble (S) fraction of rFIX proteins and empty pCold-I-coded protein after induction. Arrows indicate the location of target protein.

**Fig. 3:**
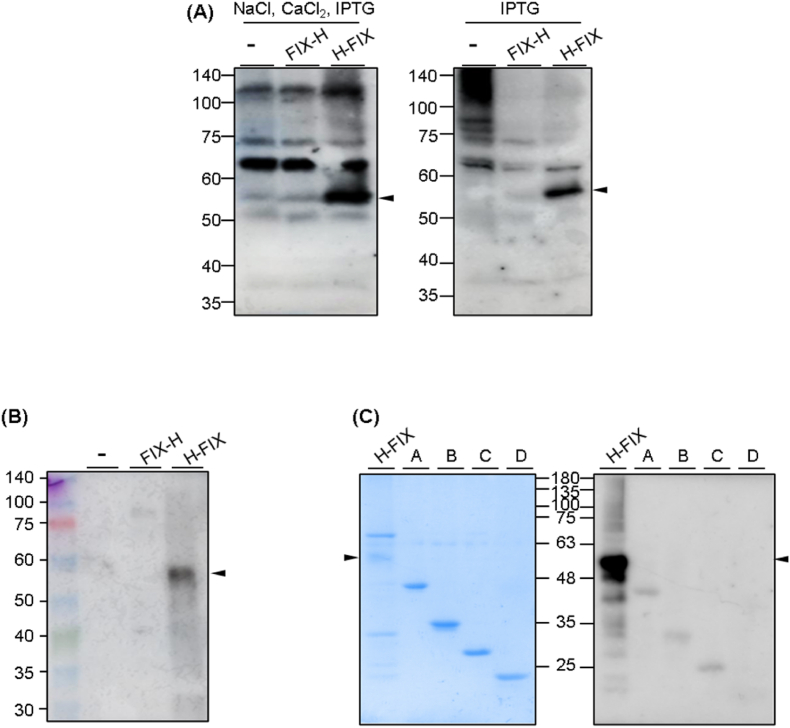
(A) Western blotting analysis of rFIX proteins and empty pCold-I-coded protein using anti-His-tag antibody; (B) western blotting analysis rFIX proteins and empty pCold-I-coded protein using anti-FIX antibody; (C) SDS-PAGE analysis and western blotting of H-FIX and four laboratory available recombinant proteins. Arrows indicate the location of target protein.

When we constructed rFIX-expressing plasmids by inserting rFIX-encoding genes into various vectors available in our lab, such as pET28a, PGK-encoding gene-inserted pET28a, and GST-encoding gene-inserted pGEX4T1, rFIX was not expressed in soluble form (Fig. 2A). Our group has generated an antibody fragment against the breast cancer marker human epidermal growth factor receptor 2 (HER2) using *E. coli*. We used pCold-I as a vector to express the anti-HER2 antibody fragment, which resulted in a high production yield (under review). Inspired by this study, we constructed the pCold-I::FIX-H and pCold-I::H-FIX plasmids and used them to express proteins. A clearer band was observed for the purified sample than for the other three samples (Fig. 1, Fig. 2C). The pCold-I vector includes a cold-shock protein A promoter for *E. coli*-based protein expression. As the vector was engineered for cold-shock expression, target proteins can be selectively induced at low temperatures by suppressing the expression of non-targeted host proteins as well as host protease activity [5,6]. These characteristics of the pCold-I vector may exert positive effects, improving the production yield of rFIXs. Notably, these proteins were expressed in soluble form. When we compared the insoluble and soluble fractions with and without IPTG induction, the target protein was observed in the induced sample (Fig. 2B and C). We conducted western blotting (WB) analysis because the target protein was barely distinguishable from the other bands on the SDS-PAGE gel. We loaded reduced rFIX proteins and an empty vector, which is a pCold-I vector without inserting an FIX-expressing gene, into an SDS-PAGE gel. We immobilized the soluble fractions onto the membrane and reacted them with an HRP-conjugated anti-His-tag antibody (Fig. 3A). A target band at approximately 53 kDa was observed for the H-FIX sample. Unexpectedly, the target band was not observed for the FIX-H sample. We performed WB using a commercial anti-FIX antibody to confirm rFIX identification (Fig. 3B and C). WB analysis was performed using a rabbit anti-FIX antibody as a secondary antibody and an HRP-conjugated anti-rabbit IgG antibody as a tertiary antibody. Similar results were obtained, in which the target protein band was not observed in non-inducing and FIX-H samples but was observed in the H-FIX sample (Fig. 3B). Although the reason for the lack of observation of the target band in the FIX-H sample is unclear, we assumed that the His-tag and FIX-H epitope may be located on the membrane side, resulting in each antibody paratope not interacting with them. We performed WB using a commercially available anti-FIX antibody against rFIX and laboratory available proteins such as Protein A (described elsewhere), Protein B (described elsewhere), *anti*-TNFa scFv [7], and BxPrx [8] to evaluate the antibody-antigen binding specificity (Fig. 3C). We observed high intensity in the target protein with a size of 53 kDa, indicating that the rFIX generated in this study had high binding activity to the anti-FIX antibody, whereas other bands appearing in the SDS-PAGE after CBB staining showed relatively low signals, indicating that the extra bands were background signals. Moreover, WB, performed using other proteins, showed that the intensity of the band signals of the non-target proteins in the rFIX sample were almost the same as those of the other samples, indicating that the other bands appearing in the SDS-PAGE analysis gel were non-specific proteins, unrelated to FIX. Our soluble proteins were purified using His-tag based affinity beads. Purity could be further increased by performing additional purification steps, such as size exclusion chromatography (SEC). However, SEC requires expensive equipment and columns, which were not available. Moreover, as we obtained sufficient quantity and protein purity for subsequent studies by single step affinity chromatography, we moved forward to the next steps in our studies using these proteins without further purification. Nevertheless, additional purification methods, such as affinity-, ion-exchange-, and/or hydrophobic/hydrophilic interaction-based purifications using various batch or chromatographic procedures, can be used to increase purity. Subsequent purification using another peptide tag, such as GST or a maltose-binding protein, can also be performed. Increasing the purity of sample for using these recombinant FIXs should be further studied. The immobilization pattern on the membrane of the antibody may differ from that on the 96-well plate because the ELISA signal was observed from FIX-H. Additionally, we attempted the addition of 0.25 M NaCl and 1 mM CaCl_2_ with IPTG, according to our recent data that showed an improved expression level of the target protein by adding NaCl and CaCl_2_ (in preparation). However, as there was no significant difference between the addition and non-addition of NaCl and CaCl_2_, only IPTG was added to the expression system. The complete procedure for generating these proteins included transformation of *E. coli* with rFIX-coding DNA, culturing cells, and induction of expression. Two days are required for transformation and cell culture, and three days are required for pCold-I-based induction [9]. Within this 5–6 day time span, the active procedure time is only 1–2 h for transformation, time for taking OD measurements during cell culture, time required to add IPTG and perform centrifugation steps. The remaining time requires no human activity. By contrast, insoluble protein expression usually requires an additional 4–5 days after induction for refolding the protein. During this additional period, the researcher must perform stepwise dialysis of urea buffer, which is time-consuming and labor-intensive.

### 3.2 Enzyme-linked immunosorbent assay

**Fig. 4:**
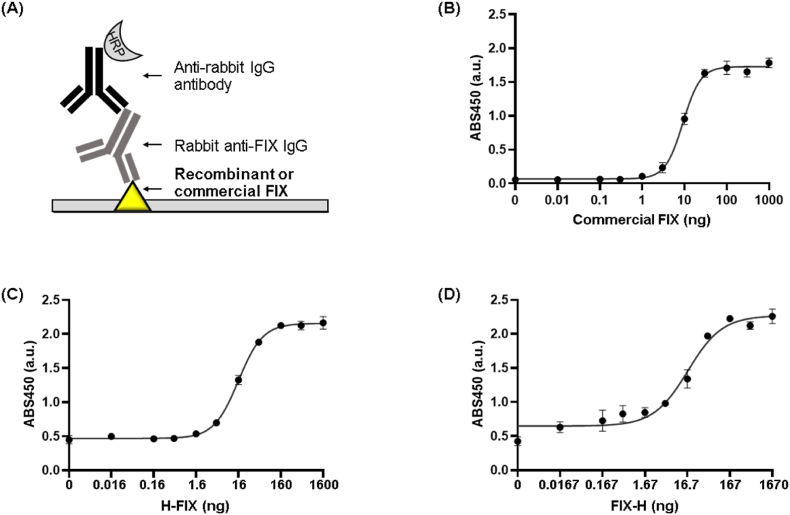
(A) Schematic representation of the ELISA; (B) ELISA signal obtained from commercial FIX; (C) ELISA signal obtained from H-FIX; (D) ELISA signal obtained from FIX-H. Error bars represent ±1 SD (n = 3). SD = standard deviation.

We conducted ELISA assays by immobilizing FIX-H or H-FIX, followed by incubation with a commercially available rabbit anti-FIX IgG, and an HRP-conjugated anti-rabbit IgG antibody (Fig. 4A). Clear dose-dependent curves were obtained from ELISAs using both FIX-H and H-FIX, indicating that both rFIXs displayed epitopes with which the anti-FIX antibody could interact (Supplementary Figure 1). The half-maximal effective concentration (EC_50_) values of FIX-H and H-FIX were 27.8 ± 2.49 and 9.90 ± 1.00 ng, respectively, and the limit of detection (LOD) values of FIX-H and H-FIX were 0.13 and 1.60 ng, respectively. We conducted ELISA using an anti-FIX antibody and commercial rFIX (Fig. 4B). We determined rFIX concentrations via ELISA using serially diluted commercial rFIX as antigens. The equation for the titration curve generated using the commercial antigen was Y = Bottom + (Top − Bottom)/(1 + 10 ((LogEC_50_-X) × Hill Slope)), where the Bottom, Top, EC_50_, and Hill Slope values were 0.06712, 1.727, 9.181, and 2.081, respectively. We substituted Y value of the equation with the absorbance intensity of 10 ng (SDS-PAGE-based concentration) FIX-H and H-FIX, 1.34 ± 0.13 and 1.32 ± 0.07, respectively, resulting in X value of 16.7 ± 3.7 and 16.0 ± 1.7 ng for FIX-H and H-FIX, respectively. At this time, we used the absorbance intensity of 10 ng protein to substitute the Y value because 10 ng was in the linear range of each curve (Fig. 4C and D).

We first estimated the rFIX concentration in the purified sample by analyzing the intensity of protein bands on SDS-PAGE using standard proteins, which is one of universal methods for quantifying protein concentration in samples containing extra proteins. However, the intensity after CBB staining sometimes does not correlate with the exact protein concentration [10,11]. Although determining the protein concentration by measuring the protein absorbance of a sample is also one of universal methods, it can be used when the purity of the protein sample is high. As the total absorbance intensity of all proteins in the sample is measured using this method, the absorbance of the target protein is not distinguishable. Accordingly, we finally determined the in-house rFIX concentration based on the ELISA titration curve generated using commercial rFIX with known concentrations. Namely, 10 ng SDS-PAGE-based calculated FIX-H and H-FIX was equivalent to 16.7 and 16.0 ng, respectively (Supplementary Figure 1).

We compared the response of commercial rFIX to anti-FIX antibody to that of in-house rFIX, resulting in lower LOD value of in-house rFIX, 0.22 ng for FIX-H and 2.24 ng H-FIX, than those of commercial rFIX, 3.19 ng. These results demonstrate that properly folded FIXs were expressed with folding and the rFIXs produced in this study can be used as antigens to capture anti-FIX IgG with high affinity and sensitivity. As mentioned above, we used rFIX samples of imperfect high purity. This was because even though contaminating non-FIX proteins could be immobilized in wells of a 96-well plate similarly to the FIXs, the primary antibody used in this study, a commercial anti-FIX antibody, would theoretically selectively recognize FIX. The extraneous proteins would not be captured to the antibody. Our ELISA results confirmed that the absorbance signals in the absence of both FIX-H and H-FIX were approximately 0.5, which might be due to non-specific binding of primary and/or secondary antibody to the wells. Absorbance signals in the presence of either rFIX increased in a protein concentration-dependent manner, demonstrating a significant interaction between protein and antibody.

## 4 Discussion

We constructed a human FIX-coding gene-inserted *E. coli* expression vector. We conventionally produced proteins in soluble form and purified them using affinity beads. We confirmed the anti-FIX antibody interaction using an ELISA. We generated two rFIX proteins with different His-tag positions because His-tag-conjugation of proteins can affect production yield and/or protein function in a location-specific manner. In our studies, no differences in yield, purity, or antibody-binding efficiency were observed, suggesting that the structures of these proteins were not disrupted by conjugating either terminal tag, and the epitopes were not oriented toward the surface of the well and were not blocked. To date, there have been no published studies on the establishment of soluble full-length rFIX without a carrier protein using *E. coli*. As PTMs are lacking in *E. coli*, most rFIXs that use the protein as a therapeutic reagent are generated using mammalian cells. However, PTMs are not essential for the use of proteins as diagnostic reagents. In this study, we confirmed that the rFIX developed herein showed a high binding affinity for the anti-FIX antibody, and its LOD was higher than that of commercial rFIX. Therefore, the rFIX can potentially be used to detect anti-FIX antibody in Hemophilia B patients, which can be applicable in pharmaceutical and medical reagents. *E. coli*-based protein expression has several merits, such as cost-effectiveness owing to the lower cost of the culture medium compared to that of mammalian cell production and the short production time, as the cell doubling time of *E. coli* is lower than that of mammalian cells. The entire procedure for producing E. coli-based rFIX was completed within five days; subculture for one day, culture for one day, induction for three days, and purification for one day. Therefore, the method proposed in this study for generating rFIX using *E. coli* could be a cost-effective tool with a short production time. We suggest the application of *E. coli*-based rFIX in which the clotting function is of no relevance, such as the use of rFIX as a protein for detecting or profiling a Hemophilia B indicator of the anti-FIX antibodies [12,13], as an inhibitor of the anti-FIX antibodies in patients with Hemophilia B [14], and as an antigen for generating a novel anti-FIX antibodies for use in FIX detection assays and Hemophilia B diagnosis [15].

## 5 Formatting of funding sources

This work was supported by the 2023 10.13039/501100013423Ottogi Grant funded by the 10.13039/501100013423Ottogi Ham TaihoFoundation, the 2020 Hemophilia Research grant funded by 10.13039/100016289Social Welfare Corporation Korea Hemophilia Foundation (2020–01), and the 10.13039/501100003725National Research Foundation of Korea (NRF) grant funded by the 10.13039/100007687Korean Government (2020R1I1A307411712).

## CRediT authorship contribution statement

**Byoung-Hee Park:** Writing – original draft, Methodology, Investigation, Funding acquisition, Formal analysis, Data curation, Conceptualization. **Hanool Yun:** Writing – original draft, Visualization, Methodology, Investigation, Data curation. **Hee-Jin Jeong:** Writing – review & editing, Visualization, Supervision, Project administration, Investigation, Funding acquisition, Conceptualization.

## Declaration of competing interest

The authors declare that they have no known competing financial interests or personal relationships that could have appeared to influence the work reported in this paper.
