# Supplementary material for: Soluble expression of recombinant coagulation factor IX protein using Escherichia coli
Source: Biochem Biophys Rep. 2024 Apr 18;38:101714. doi: 10.1016/j.bbrep.2024.101714 (PMC11052911; doi:10.1016/j.bbrep.2024.101714)
Supplement: Multimedia component 1 [file mmc1.docx]

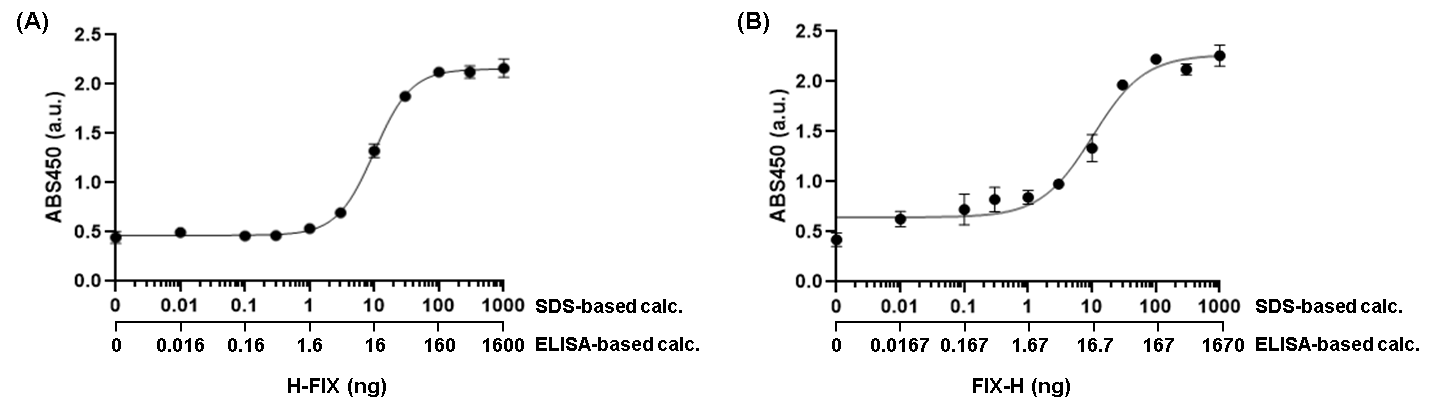


**Supplementary Figure 1.** (A) ELISA signal obtained from H-FIX; (B) ELISA signal obtained from FIX-H. calc. indicates calculation.
